# Functional Silsesquioxanes—Tailoring Hydrophobicity and Anti-Ice Properties of Polylactide in 3D Printing Applications

**DOI:** 10.3390/ma17194850

**Published:** 2024-10-01

**Authors:** Roksana Konieczna, Robert E. Przekop, Daria Pakuła, Julia Głowacka, Katarzyna Ziętkowska, Rafał Kozera, Bogna Sztorch

**Affiliations:** 1 Centre for Advanced Technologies, Adam Mickiewicz University Poznan, Uniwersytetu Poznańskiego 10, 61-614 Poznan, Poland; roksana.konieczna@amu.edu.pl (R.K.); rprzekop@amu.edu.pl (R.E.P.); darpak@amu.edu.pl (D.P.); julia.glowacka@amu.edu.pl (J.G.); 2 Faculty of Chemistry, Adam Mickiewicz University in Poznań, Uniwersytetu Poznańskiego 8, 61-614 Poznan, Poland; 3 Faculty of Materials Science and Engineering, Warsaw University of Technology, ul. Woloska 141, 02-507 Warszawa, Poland; katarzyna.zietkowska.dokt@pw.edu.pl (K.Z.); rafal.kozera@pw.edu.pl (R.K.)

**Keywords:** polylactide, 3D printing, hydrophobic materials, organosilicon compound

## Abstract

To explore the tailoring of hydrophobicity in 3D-printed polylactide (PLA) composites for advanced applications using additive manufacturing (AM), this study focuses on the use of Fused Deposition Modeling (FDM) 3D printing. PLA, a material derived from renewable sources, is favored for its eco-friendliness and user accessibility. Nonetheless, PLA’s inherent hydrophilic properties result in moisture absorption, negatively affecting its performance. This research aims to modify PLA with organosilicon compounds to enhance its hydrophobic and anti-icing properties. Incorporating fluorinated siloxane derivatives led to significant increases in water contact angles by up to 39%, signifying successful hydrophobic modification. Mechanical testing demonstrated that the addition of organosilicon additives did not compromise the tensile strength of PLA and, in some instances, improved impact resistance, especially with the use of OSS-4OFP:2HEX:2TMOS, which resulted in an increase in the tensile strength value of 25% and increased impact strength by 20% compared to neat PLA. Differential scanning calorimetry (DSC) analysis indicated that the modified PLA exhibited reduced cold crystallization temperatures without altering the glass transition or melting temperatures. These results suggest that organosilicon-modified PLA has the potential to expand the material’s application in producing moisture and ice-resistant 3D-printed prototypes for various industrial uses, thereby facilitating the creation of more durable and versatile 3D-printed components.

## 1. Introduction

Additive manufacturing (AM), also known as 3D printing, has gained significant attention in various specialized fields due to advancements in material science. Three-dimensional printing allows the production of parts with personalized shapes, making it possible to produce parts with shapes that are inaccessible to common manufacturing methods [1,2]. One of the prominent methods in this field is Fused Deposition Modeling/Filament Fused Fabrication (FDM/FFF) 3D printing, which uses thermoplastic polymers, with polylactide (PLA) being the primary choice [3,4]. PLA, derived from renewable sources like corn starch or sugarcane, is valued for its eco-friendly properties and biodegradability [4]. With its low melting point, PLA enables smooth printing processes, making it popular for its user-friendly characteristics, excellent surface finish, and ability to create intricate prints [5]. However, the hydrophilic nature of PLA introduces a susceptibility to ambient moisture absorption, impairing the material’s handling and performance in 3D printing applications [6].

In recent years, much attention has been given to solving the problem of icing on various surfaces and structures, which causes a lot of performance and economic and social damage in the industry [7]. One of the methods known from the literature is to obtain ice-phobic (anti-icing) surfaces [8]. These surfaces should be characterized, among other things, by the lowest possible values of the adhesion strength between the substrate and the ice, so that the resulting ice could be detached under the action of natural forces like wind or gravity [9]. The mechanism for developing such surfaces can be to modify them with compounds that lower surface energy. Surfaces exhibiting a hydrophobic nature can reduce the time and area of contact between subcooled water droplets and the substrate and consequently increase anti-icing performance [10]. Organosilicon compounds with appropriately selected functional groups, such as alkyl or fluoroalkyl groups, can significantly influence the surface properties, including the hydrophobicity, of materials. [11]. It has been proven that chemical modification with such compounds can lead to an increase in surface water contact angles up to about 120° [7]. However, in addition to chemical modification, various other surface treatment techniques are commonly applied to 3D-printed plastics to further enhance their properties. These methods include mechanical abrasion, plasma treatment, thermal processes, and UV exposure. For instance, plasma discharge methods are employed to enhance adhesion between material layers, as adhesion is a critical factor in the quality of 3D prints. One promising approach is the use of diffuse coplanar surface barrier discharge (DCSBD) plasma, which significantly improves adhesion in polylactic acid (PLA) materials [12]. Likewise, thermal treatments can improve mechanical strength and promote better layer adhesion by relieving internal stresses [13]. UV treatment, particularly UV-excimer laser irradiation, has also been shown to effectively modify polymer surfaces by altering surface chemistry, enhancing adhesion, and enabling crosslinking and cleaning [14].

In response to the need to develop materials with anti-icing and superhydrophobic properties, organosilicon compounds offer a promising route to increase the resistance of polylactide filaments used in AM to moisture absorption [15,16]. Furthermore, this modification holds promise in broadening PLA’s utility spectrum, enabling its application in the fabrication of 3D-printed prototypes for aerodynamic components resistant to moisture and ice deposition in a one-step method without special coatings [17,18]. Although multiple barriers overlap with superhydrophobic materials and coatings, limiting their application in industry and daily life, the development of superhydrophobic surfaces still holds significant potential across various fields [19]. For instance, 3D-printed superhydrophobic surfaces can play a pivotal role in developing surgical instruments with heightened resilience against biological contaminants, thereby improving sterilization efficacy and raising hygiene standards [20]. Custom superhydrophobic channels can be applied in microfluidic systems, enabling precise fluid flow control without the risk of contamination or fluid retention [21]. Furthermore, integrating superhydrophobic structures into textile substrates can create personalized clothing that repels water and ice, particularly for sports and outdoor apparel [22,23]. Moreover, the effectiveness of superhydrophobic surfaces in preventing ice adhesion has important implications for industries such as aviation and renewable energy, including wind turbines [24,25].

Organosilicon compounds include such compounds as silanes, silsesquioxanes, and polysiloxanes. Silsesquioxanes are composed of building units Si-O-Si with a general chemical formula (RSiO_1.5_)n, where R can denote any type of organic group or hydrogen [26]. The presence of highly reactive Si-H bonds enables the functionalization of compounds through hydrosilylation reactions with olefins containing diverse functional groups, tailored to the requirements of the final material, which allows for extensive control over the properties of modified polymer surfaces. Polyhedral oligomeric silsesquioxane (POSS) used for hydrophobic and superhydrophobic surfaces had shown great promise [27], so many POSS-containing anti-icing coatings were developed over the years. The results showed that POSS increased surface roughness and decreased surface energy simultaneously, resulting in low ice adhesion strength values [16,28,29]. Other works also proved the positive hydro- and ice-phobic effects of organosilicon compounds [11].

This article presents the results of a study on the modification of PLA by the addition of fluorinated spherosilicate derivatives. The new composites were obtained in 3D printing technology and were subjected to detailed analysis, including melt flow index, surface analysis, and mechanical testing, to evaluate the improvement of their functional properties. The use of optical microscopy enabled the understanding of the complex morphology of the obtained composites and its impact on their mechanical properties. Additionally, thermal stability tests of the obtained samples were conducted using thermogravimetric analysis (TGA), and their thermal transitions were assessed through differential scanning calorimetry (DSC). Furthermore, the contact angle and the ice adhesion properties of the new composites with fluorinated spherosilicate derivatives were evaluated.

## 2. Materials and Methods

### 2.1. Materials

Polylactide (PLA) Ingeo 2003D type was purchased from NatureWorks (Minnetonka, Minneapolis, MN, USA). The reagents for chemical synthesis were purchased from the following sources: hexene (HEX) from Thermo Scientific Chemicals (Waltham, MA, USA); vinyl trimethoxysilane (TMOS) from ABCR (Karlsruhe, Germany); toluene, chloroform-d, Karstedt’s catalyst in xylene solution from Merck Group (Darmstadt, Germany). Allyl 2,2,3,3,4,4,5,5-octafluoropentyl ether (Allyl-OFP) was synthesized according to the literature [30]. Octaspherosilicate (OSS) was synthesized according to the literature [31].

### 2.2. Analyses

Fourier transform infrared (FTIR) spectra were recorded on a Nicolet iS 50 Fourier transform spectrophotometer (Thermo Fischer Scientific) equipped with a diamond ATR unit with a resolution of 0.09 cm^−1^.

^1^H, ^13^C, and ^29^Si nuclear magnetic resonance (NMR) spectra were recorded at 25 °C on Bruker Ascend 400 and Ultra Shield 300 spectrometers using CDCl_3_ as a solvent. Chemical shifts are reported in ppm concerning the residual solvent (CHCl_3_) peaks for ^1^H and ^13^C.

The melt flow rate (MFR) was measured using the Instron CEAST MF20 melt flow tester according to the standard EN ISO 1133 [32] at 210 °C for the load of 2160 g and the time of cutting off the polymer stream was 15 s.

Water contact angle (WCA) analyses were performed by the sessile drop technique at room temperature and atmospheric pressure with a Krüss DSA100 goniometer. Three independent measurements were taken for each sample, each with a 5 µL water drop, and the obtained results were averaged.

Mechanical tensile tests were conducted using the universal testing machine INSTRON 5969 with a maximum measuring capability of 50 kN. For tensile strength tests, standard 1BA specimens were used following the requirements of PN-EN ISO 527, [33] and for the flexural test specimens were prepared by PN-EN ISO 178, [34]. For all the series, 7 measurements were performed. The average and standard deviation were determined for each measurement series. The traverse speed for the tensile strength measurements was set at 5 mm/min.

A Charpy impact test (with no notch) was performed on an Instron Ceast 9050 impact machine according to ISO 179-135, [35].

Thermogravimetry (TGA) was performed using a NETZSCH 209 F1 Libra gravimetric analyzer (Selb, Germany). 9 mg ± 0.5 mg samples were cut and placed in Al_2_O_3_ crucibles. Measurements were conducted under nitrogen (flow of 20 mL/min) in temperature ranges from 30 °C to 800 °C and at a 10 °C/min heating rate.

Differential scanning calorimetry (DSC) was performed using a NETZSCH204 F1 Phoenix calorimeter. Samples of 6 mg ± 0.2 mg were cut from each granulate and placed in an aluminum crucible with a punctured lid. The measurements were performed under nitrogen in the 20–220 °C temperature range and at a 10 °C/min heating rate.

**Figure 1:**
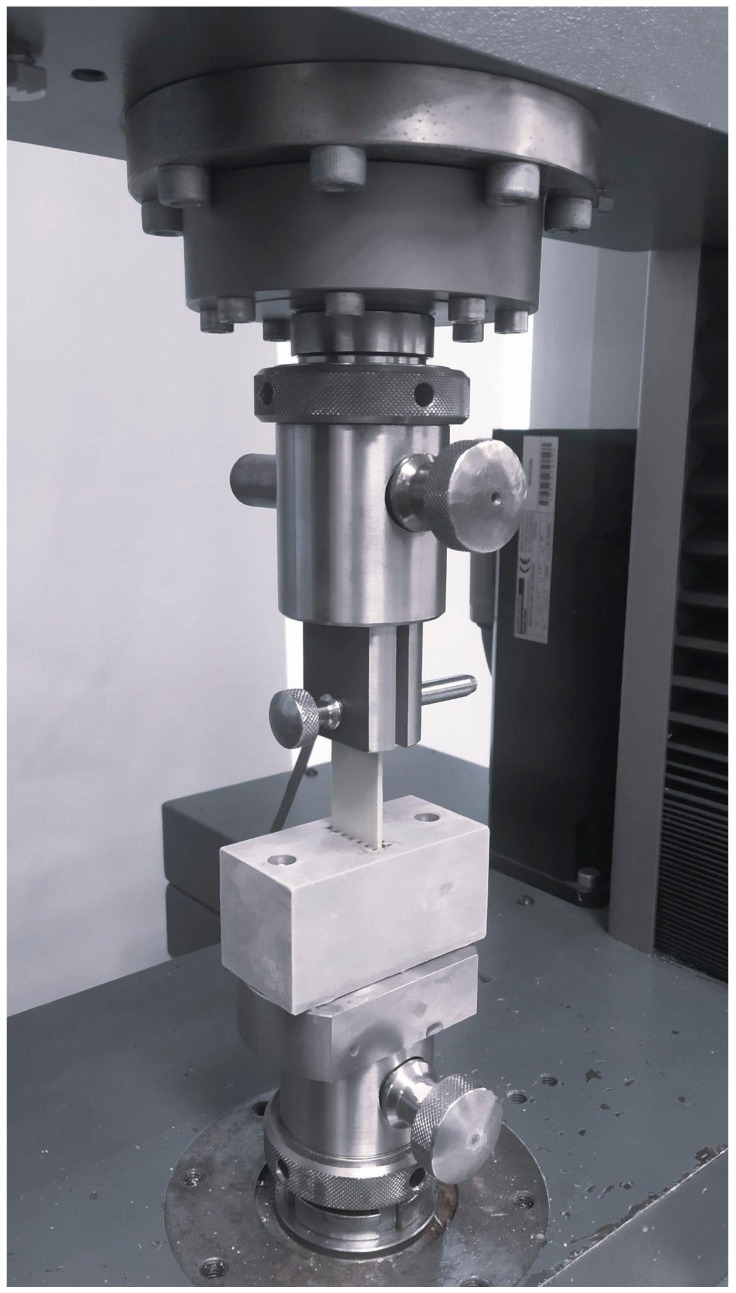
The ice adhesion testing machine with a mounted sample.

The ice adhesion strength (IA) was performed using a universal tensile testing machine, Zwick/Roel Z050, by shearing the ice layer (Figure 1). A maximum force was measured until the frozen sample was removed from the holder. The ice adhesion is the maximum force related to the contact area between the ice and the sample surface. The measurement method was presented in detail in previous work [36].

Light microscopy images of the fractures of the composites were taken using a KEYENCE VHX-7000 digital microscope (Keyence International, Mechelen, Belgium, NV/SA) with a 100–1000 VH-Z100T zoom lens. All images were captured using a VHX 7020 camera at 100× magnification.

### 2.3. The Procedure for Synthesis of Spherosilicate-Based Derivatives

In a conventional procedure, a 500 mL three-necked round-bottom flask is loaded with 20 g of OSS, 250 mL of toluene, and the calculated amount of olefin (Table 1), and a magnetic stir bar is added. The reaction mixture was adjusted to 70 °C. Before the boiling point was reached, the Karstedt catalyst solution (8 × 10^−5^ eq Pt/mol SiH) was added, causing the temperature to rise rapidly and the system to start refluxing. The reaction mixture was kept in reflux and samples were taken for FTIR control until complete consumption of the Si-H group was observed (based on the disappearance of the characteristic signals corresponding to the stretching and bending vibrations of the Si-H group, observed at 2141 cm^−1^ and 889 cm^−1^, respectively). The solvent was then evaporated under a vacuum to dryness to obtain an analytically pure sample.

### 2.4. Preparation of PLA/OSS Composite Masterbatches

The PLA/OSS composite system was obtained using a ZAMAK MERCATOR WG 150/280 laboratory two-roll mill (Zamak Mercator, Skawina, Poland). A portion of 475 g of PLA Ingeo™ 2003 D was mixed with 25 g OSS-based derivative until the final concentration of the additive reached 5% *w*/*w*. The ingredients were mixed for 15 min at 215 °C, and a roller speed of 20 rpm. This process was performed until the ingredients were completely homogenized. The resulting polymer system was then granulated with a SHINI SG-1417-CE grinder (Shini Plastics Technologies, Taichung, Taiwan) and dried at 55 °C for 24 h.

### 2.5. Preparation of Filament

The masterbatches were ground and extruded into a filament for 3D printing. The masterbatch and neat PLA were diluted to 6 final concentrations: 0.1%, 0.25%, 0.5%, 1%, 1.5%, and 2.5%. The filament was extruded on a Filabot EX6 (Filabot, Barre, VT, USA) single-screw extruder with four heating zones, an L/D 24 screw, and a nozzle with a diameter of 1.75 mm.

### 2.6. 3D Printing (FDM)

The extruded filament was used for FDM (Fused Deposition Modeling) 3D printing. Using a 3D printer, Prusa i3 MK3S +, two types of samples were printed by FDM: paddles and bars, according to PN-EN-ISO 527−2 [37]. Parameters of printing are given in Table 2. Bars for impact and bending tests were printed and paddles for stretching tests.

## 3. Results and Discussion

### 3.1. Chemical Characterization of Modifiers

**Figure 2:**
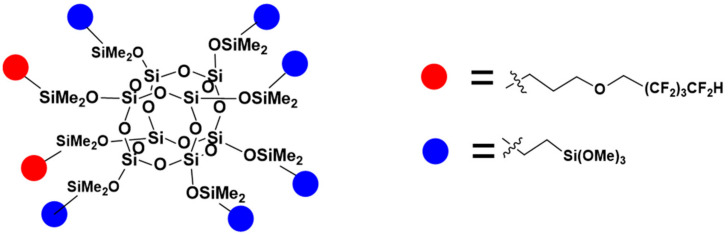
The formula of OSS-2OFP:6TMOS.

**Figure 3:**
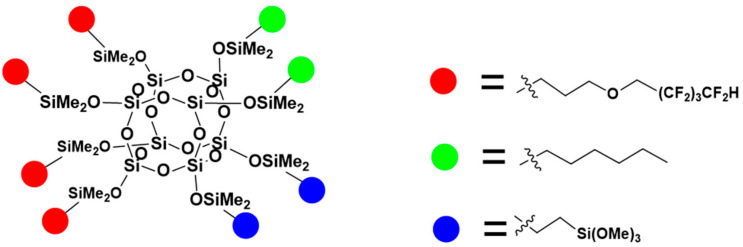
The formula of OSS-4OFP:2HEX:2TMOS.

**Figure 4:**
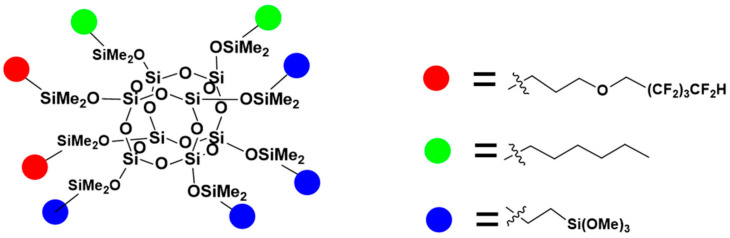
The formula of OSS-2OFP:2HEX:4TMOS.

The hydrosilylation reactions of octaspherosilicate (OSS) with different types of olefins (Figure 2, Figure 3 and Figure 4) were conducted until the attenuation of the characteristic signal corresponding to the Si-H moiety at approximately 2100 cm^−1^, indicating complete conversion of the OSS. Vinyltrimethoxysilane, allyl 2,2,3,3,4,4,5,5-octafluoropentyl ether, and alkenes with varying carbon chain lengths, such as hexene, were used as olefins, attached to the core in different molar ratios. The reaction products were obtained with high yields of ~ 95%. Comprehensive NMR analysis encompassing ^1^H, ^13^C, and ^29^Si spectra were employed to corroborate the synthesized compounds’ structural integrity, purity, and conversion efficiency.

**Figure 5:**
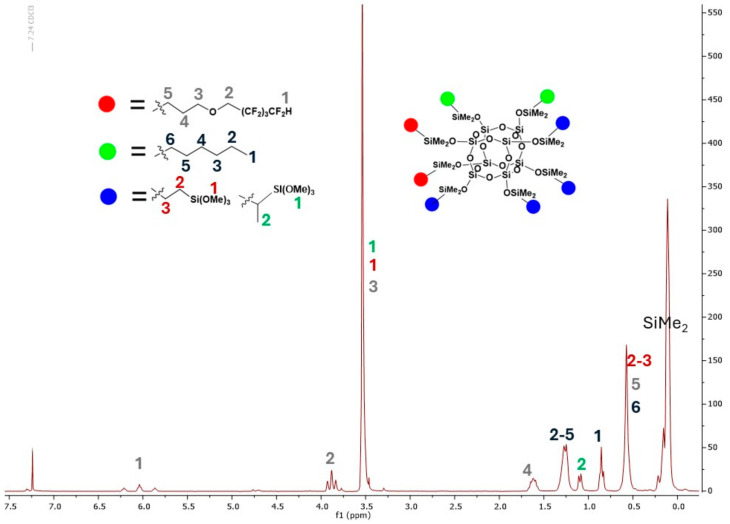
The NMR spectrum of OSS-2OFP:2HEX:4TMOS.

Figure 5 presents the proton NMR spectrum of the selected compound in deuterated solvent, CDCl₃. Based on chemical shifts and integration, individual signals were assigned to the corresponding structural protons, confirming consistency with the proposed structure of compound OSS-2OFP:2HEX:4TMOS.

^1^H NMR (400 MHz, CDCL_3_): σ(ppm) = 6.25–5.87 (tt, J1 = 52.2Hz, J¬2 = 5.6Hz, 2H, -CF_2_H), 3.90 (t, J = 14.1Hz, 4H, O CH_2_-CF_2_), 3.55–3.51 (m, 58H, OMe, O-CH_2_-CH_2_-CH_2_-Si), 1.68–1.58 (m, 4H, O-CH_2_-CH_2_-CH_2_-Si), 1.10 (d, J = 7.5Hz, alpha product -CH_3_), 0.62–0.57 (m, 28H, O-CH_2_-CH_2_-CH_2_-Si, SiCH_2_CH_2_Si), 0.17, 0.14, 0.13, 0.12, 0.11 (s, 48H, SiMe_2_) ^13^C NMR (101 MHz, CDCl_3_): σ(ppm) = 117.90 (CF_2_H, OFP), 115.38, 110.06, 107.45, 104.92 (CH_2_, CF_2_), 74.68 (O-CH_2_-CH_2_-CH_2_-Si), 67.44 (t, O-CH_2_-CF_2_), 49.88, 23.08 (O-CH_2_-CH_2_-CH_2_-Si), 12.84 (O-CH_2_-CH_2_-CH_2_-Si), 8.45, 0.42 (Si-CH_2_CH_2_-Si), −0.39 (fluorinated group SiMe_2_), −1.04 (trimethoxysilylethyl group SiMe_2_);^29^Si NMR (79,5 MHz, CDCl_3_): σ(ppm) = 13.52–13.28 (SiMe_2_), −42.90 (Si(OMe)_3_), −109.02 (cage).

^1^H NMR (400 MHz, CDCL_3_): σ(ppm) = 6.25–5.86 (tt, J1 = 52.2Hz, J¬2 = 5.6Hz, 4H, -CF_2_H), 3.95–3.85 (t, J = 14.1Hz, 8H, O-CH_2_-CF_2_), 3.56–3.52 (m, 26H, OMe, O-CH_2_-CH_2_-CH_2_-Si), 1.70–1.59 (m, 8H, O-CH_2_-CH_2_-CH_2_-Si), 1.32–1.26 (m, 16H, hexyl -CH_2_- groups), 1.14–1.10 (m, alpha product -CH_3_), 0.90–0.56 (m, 6H, hexyl -CH_3_), 063–0.57 (m, 20H, O-CH_2_-CH_2_-CH_2_-Si, SiCH_2_CH_2_Si), 0.14, 0.11 (s, 48H, SiMe_2_); ^13^C NMR (101 MHz, CDCl_3_): σ(ppm) = 117.87 (CF_2_H, OFP), 115.37, 110.00, 106.45, 105.21 (CH_2_, CF_2_), 77.55 (O-CH_2_-CH_2_-CH_2_-Si), 68.33 (t, O-CH_2_-CF_2_), 54.28, 22.99 (O-CH_2_-CH_2_-CH_2_-Si), 33.36, 30.85, 28.28, 25.58, 23.00, 15.52, 11.42 (hexyl), 12.89 (O-CH_2_-CH_2_-CH_2_-Si), 8.40, 0.34 (Si-CH_2_CH_2_-Si), −0.35 (fluorinated group SiMe_2_), −1.00 (trimethoxysilylethyl group SiMe_2_);^29^Si NMR (79,5 MHz, CDCl_3_): σ(ppm) = 13.38–12.77 (SiMe_2_), −41.26 (Si(OMe)_3_), −108.45 (cage).

^1^H NMR (400 MHz, CDCL_3_): σ(ppm) = 6.17–5.91 (m, 2H, -CF_2_H), 3.91 (t, J = 14.1Hz, 4H, O-CH_2_-CF_2_), 3.56–3.50 (s, OMe, 36H; CH2-CH2-CH2-O, 4H), 1.66–1.59 (m, 4H, O-CH_2_-CH_2_-CH_2_-Si), 1.28–1.26 (m, 16H, hexyl -CH_2_- groups), 1.11 (d, J = 7.5Hz, 3H, alpha product -CH_3_), 0.87–0.84 (t, 6H, -CH-_2_CH_3_), 0.54 (m, 24H, -CH_2_-CH_2_-CH_2_-Si, SiCH_2_CH_2_Si), 0.15, 0.14, 0.12 (s, 48H, SiMe_2_);^13^C NMR (101 MHz, CDCl_3_): σ(ppm) = 117.89 (CF_2_H, OFP), 115.41, 110.02, 107.49, 104.95 (CH_2_, CF_2_), 75.46 (O-CH_2_-CH_2_-CH_2_-Si), 67.33 (t, O-CH_2_-CF_2_), 50.33, 22.79 (O-CH_2_-CH_2_-CH_2_-Si), 34.40, 31.52, 29.20, 26.09, 22.23, 15.82, 12.6 (hexyl), 13.21 (O-CH_2_-CH_2_-CH_2_-Si), 8.42, 0.39 (Si-CH_2_CH_2_-Si), −0.37 (fluorinated group SiMe_2_), −1.02 (trimethoxysilylethyl group SiMe_2_);^29^Si NMR (79,5 MHz, CDCl_3_): σ(ppm) = 13.31–12.68 (SiMe_2_), −40.99 (Si(OMe)_3_), −108.40 (cage).

### 3.2. Microscopy

**Figure 6:**
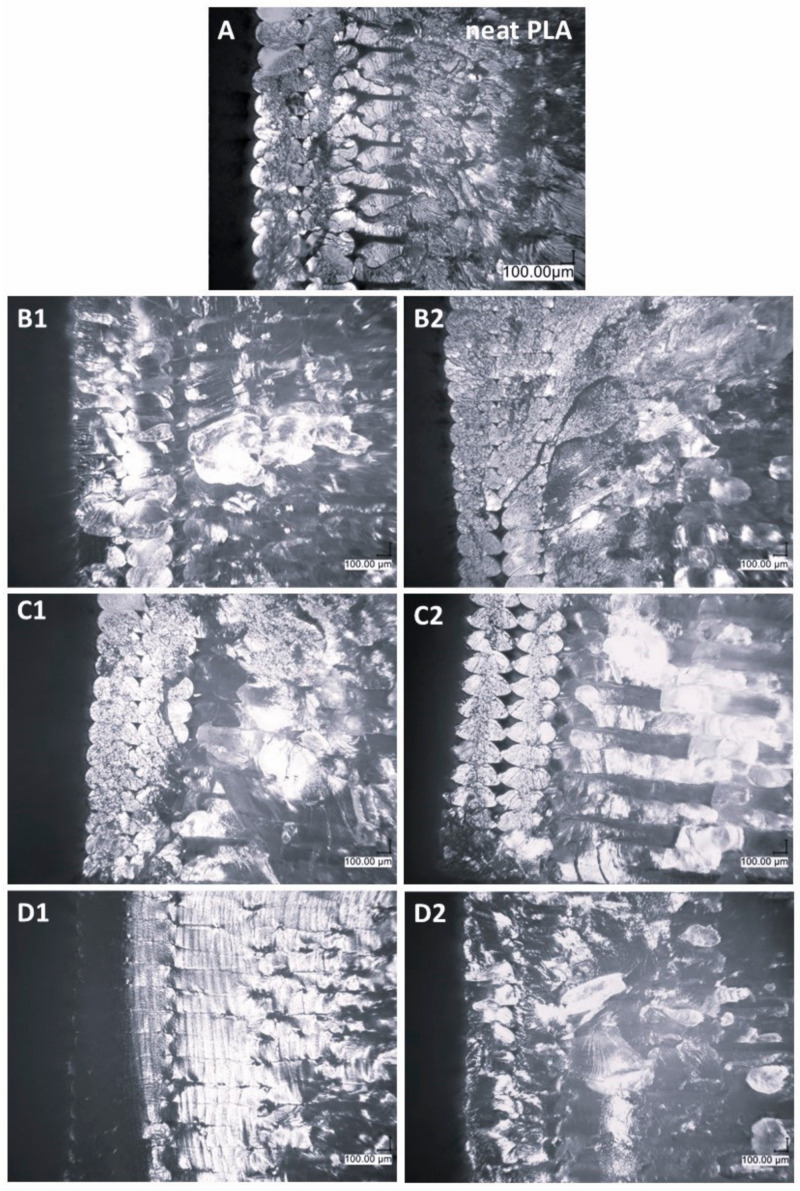
Optical microscope images of cross-sections of printed PLA samples after impact testing of (**A**) neat PLA, (**B1,B2**) OSS-2OFP:6TMOS, (**C1,C2**) OSS-2OFP:2HEX:4TMOS, (**D1,D2**) OSS-4OFP:2HEX:2TMOS; modification concentration 1–0.25% and 2–1.5%.

Figure 6 shows the fractures of 3D-printed samples after being subjected to Charpy impact testing. Microscopic images of the fracture surfaces of samples obtained through Fused Deposition Modeling (FDM) technology depict the coherence between individual layers of the material. They also reveal spaces that may exist between these layers, providing insight into any potential irregularities or voids. These images serve as a valuable tool for examining the internal structure of the printed samples, allowing for the identification of anomalies or deficiencies in the layer-to-layer cohesion.

In the reference PLA sample, the interlayer bonding is less strong than in most of the modified samples, exhibiting lower degrees of layer-to-layer cohesion. Significantly, the OSS-4OFP:2HEX:2TMOS composite (Figure 6D1) exhibits superior interlayer fusion compared to both the reference PLA and other modified samples. This suggests that the specific combination of organic modifiers in OSS-4OFP:2HEX:2TMOS improves the overall structural integrity of the printed material by reducing voids and increasing layer adhesion. The largest spaces, both between the external outlines and between the outline and the infill of the sample, are present in the OSS-2OFP:2HEX:4TMOS composite (Figure 6C2). For most systems, an increased amount of free air voids is observed in composites with higher concentrations of the modifier.

### 3.3. Thermal Analysis Results

Thermogravimetric analysis (TGA) was conducted in a flow of inert nitrogen gas for neat polymer and modified materials with the highest concentration of organosilicon compound, i.e., 1.5% by weight, to determine potential changes occurring during decomposition under temperature influence. From the obtained thermogravimetric (TGA) and differential thermogravimetric (DTG) curves, temperatures corresponding to 1% and 5% mass loss (T_1%_ and T_5%_), onset decomposition temperature (Tonset), and temperature at the maximum decomposition rate (T_max_) were determined (Table 3).

**Figure 7:**
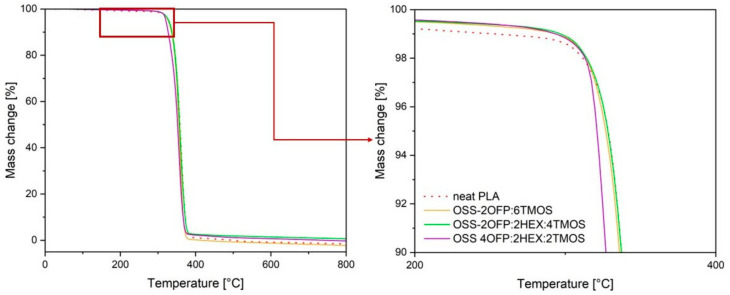
TGA curves of PLA composites in a nitrogen atmosphere.

**Figure 8:**
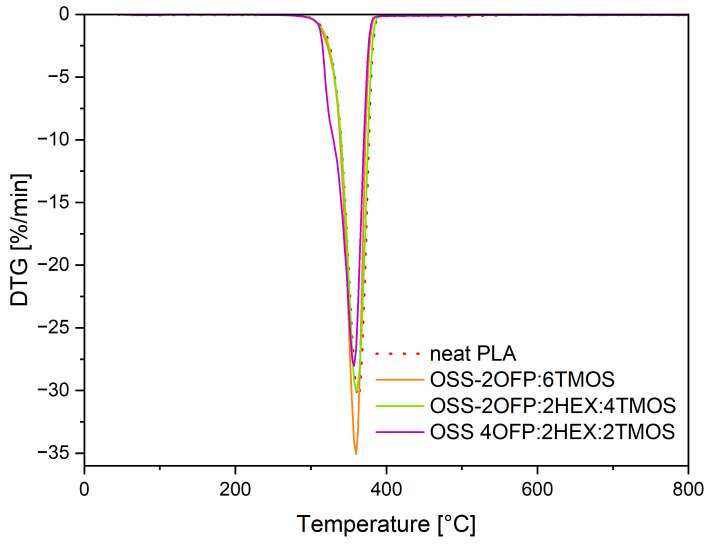
DTG curves of PLA composites in a nitrogen atmosphere.

Based on the TGA curves (Figure 7), it was observed that each material exhibited a single-step thermal decomposition. Based on TGA and DTG (Figure 8), for the temperature corresponding to 1% mass loss, composites exhibited higher values compared to neat PLA (T_1%_ = 253.2 °C), indicating a lower amount of volatile products (including moisture and impurities) in the modified materials. For the parameters T_5%_, T_onset_, and T_max_, the values for PLA and all composites were similar and ranged as follows: T_5%_ = 321–329 °C, T_onset_ = 336–343 °C, T_max_ = 358–363 °C. The obtained results confirm that the introduced organosilicon modifiers into PLA did not significantly alter the main stages of the polymer’s thermal decomposition, indicating that these modifications did not destabilize the polymer structure in a way that would affect its thermal stability. Modifying polylactide by adding modifiers with selected functional groups may lead to materials with improved usability properties while maintaining their thermal characteristics.

### 3.4. Differential Scanning Calorimetry (DSC)

**Figure 9:**
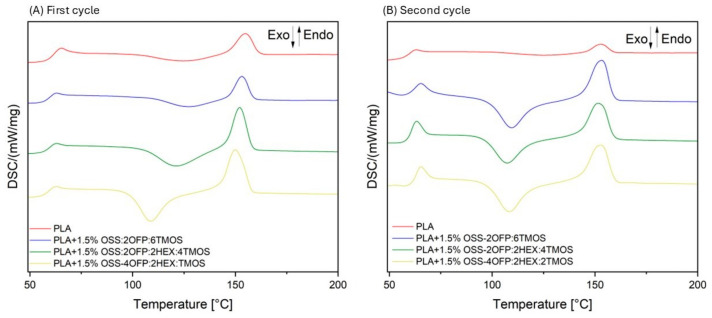
DSC curves of PLA and composites.

Differential scanning calorimetry (DSC) analysis was conducted on neat PLA and modified systems to observe the phase transitions occurring in the polymer and the influence of organosilicon modifiers on these processes. Figure 9A,B present the thermograms recorded during the first and second heating cycles. Based on the obtained curves, characteristic phase transitions typical for semicrystalline polymers were identified, such as the glass transition temperature (T_g_) in the range of 55–70 °C, cold crystallization temperature (T_cc_) in the range of 90–130 °C, and melting temperature (T_m_) in the range of 135–160 °C. For neat PLA, these values are T_g_ = 64.7 °C (first cycle) and 62.4 °C (second cycle), T_cc_ = 124.3 °C (first cycle) and 123.1 °C (second cycle), and T_m_ = 155.2 °C (first cycle) and 153.2 °C (second cycle) (Table 4).

For the modified samples, no significant changes in glass transition temperature (T_g_) and melting temperature (T_m_) are observed compared to the neat PLA sample. For all systems in the first heating cycle, lower cold crystallization temperatures (T_cc_) are recorded, lower by up to 16.1 °C. In the second heating cycle, a shift towards lower temperatures is observed for each composite system, ranging from 14.1 °C to 16.1 °C. The reduction in cold crystallization temperatures is associated with the increased amount of OFP and alkyl groups in the organosilicon modifiers introduced into the PLA matrix. The lower cold crystallization temperature in composites achieved through the addition of organosilicon compounds is attributed to their function as nucleating agents.

Using Equation (1), the degree of crystallinity for the composites was determined for both the first and second thermal cycles. In the initial cycle, polylactide exhibited a crystallinity of 14.5%, which decreased to 5.4% in the subsequent cycle. The inclusion of organosilicon compounds was found to significantly influence the thermal behavior of the composites. Specifically, these additives reduced the cold crystallization temperature. Additionally, in the case of the composite modified with 1.5% OSS-2OFP:6TMOS, the degree of crystallinity in the second cycle increased remarkably to 28.9%. This indicates that the presence of organosilicon compounds facilitates better nucleation, leading to improved crystalline structure in the modified PLA composites.

(1) $$w_{c}=\frac{{\Delta H}_{m}}{{\Delta H}_{m}^{0}}\times 100\%$$

*w_c_*—degree of crystallinity, ∆*H_m_*—heat of fusion, ∆*H_m_*^0^—heat of fusion of 100% crystalline polymer; for neat PLA = 93.6 J/g [38].

### 3.5. Water Contact Angle (WCA)

**Figure 10:**
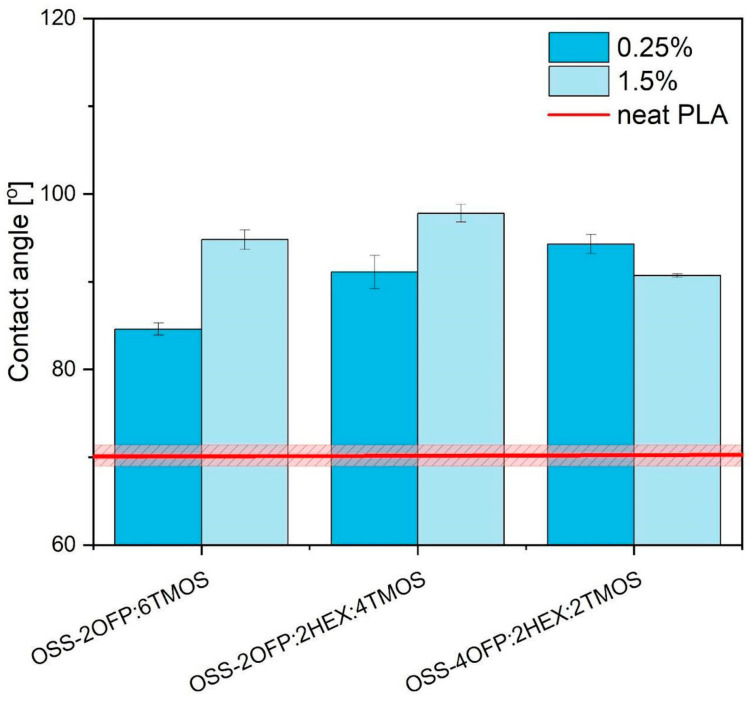
The water contact angle for all samples.

Analysis was conducted to measure the water contact angle (WCA) for a series of polylactic acid (PLA)-based composites (Figure 10). The unmodified polymer, characterized by its hydrophilic properties, exhibited a water contact angle of 70.2° (Table 5), indicating its limited resistance to water exposure. The introduction of silsesquioxane (OSS) derivatives into the PLA matrix resulted in a significant alteration of the surface properties of the composites towards hydrophobic properties. The addition of selected modifiers led to an increase in the contact angle ranging from 14.4° to 27.6°, depending on the type and concentration of the OSS derivative used.

Alkyl groups introduced into the PLA matrix interact weakly with water through dispersion forces. In contrast, fluoroalkyl groups, which possess higher hydrophobic properties and additional oleophobicity, demonstrate significantly greater effectiveness in repelling both water and oils [39,40,41]. The increase in the water contact angle of PLA-based composites due to the use of functionalized silsesquioxane derivatives has significant practical applications. Such changes in surface characteristics can substantially impact the future applications of materials in industries requiring high water resistance. The effectiveness of these modifiers in enhancing hydrophobic and oleophobic properties may also contribute to the development of new composite materials with specialized functionalities, thus opening new avenues for advanced industrial and technological applications.

### 3.6. Ice Adhesion

**Figure 11:**
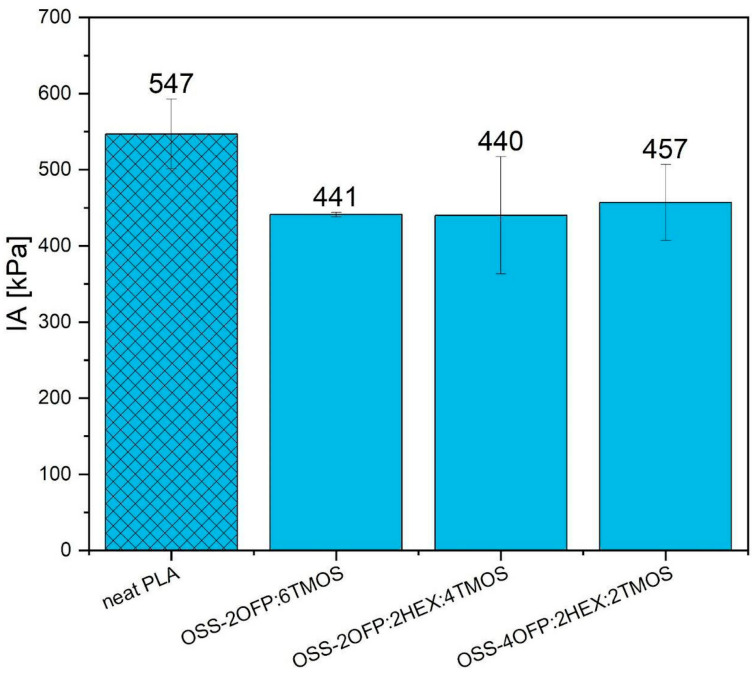
Ice adhesion for all samples.

The ice adhesions obtained for the tested samples—one of the parameters determining the anti-icing performance of the surface—are shown in Figure 11. The reference sample, pure PLA, achieved the IA of 547 kPa. For composites modified with functionalized spherosilicates, the range of values for this parameter was from 440 to 457 kPa. All samples recorded a reduction in IA compared to pure PLA. The lowest IA, i.e., the highest improvement, was obtained by the OSS-2OFP:2HEX:4TMOS composite, with a reduction in value of 20% compared to the unmodified sample. However, it can be concluded that all modified surfaces achieved similar IA. The works [11,42] also proved the positive effect of organosilicon compounds on the ice-phobic properties of polymer matrix surfaces.

### 3.7. Mechanical Performance

This section discusses the results of PLA modification with organosilicon compounds on the mechanical performance of PLA composite 3D-painted samples.

#### 3.7.1. Tensile Test Results

**Figure 12:**
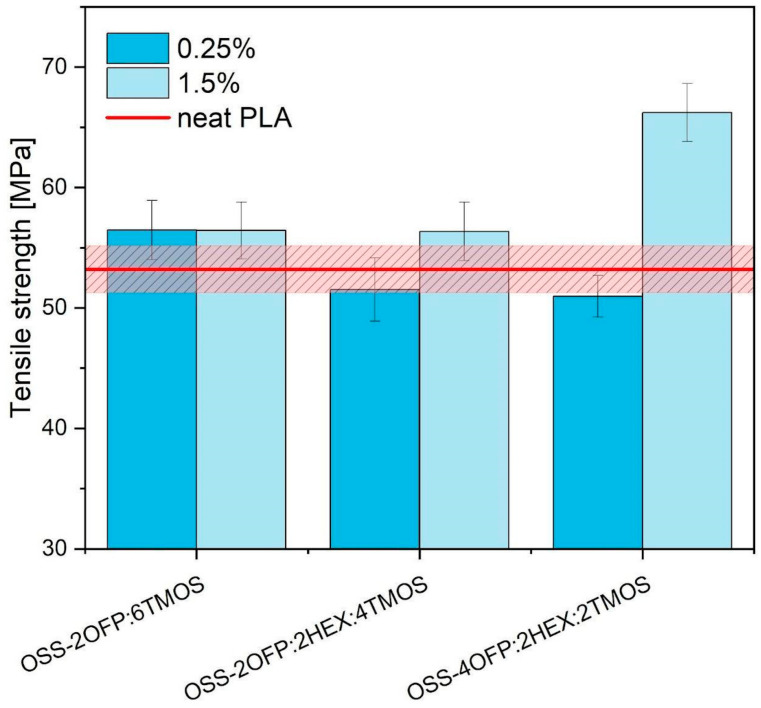
Tensile strength of PLA samples in 3D printing.

Tensile tests were performed to describe the strain–tensile behavior of 3D-printed PLA composite materials. Figure 12 presents the influence of different di- and trifunctional organosilicon additives on PLA tensile strength. The effect of modifier concentration on the toughness of the modified PLA was also determined. PLA Ingeo 2003D tensile strength for 3D-printed samples is approximately 53.22 MPa ± 1.9 MPa. Based on the presented data, organosilicon compounds did not negatively affect the PLA tensile strength. Various scientific research confirms the positive effect of organosilicon compounds including silsesquioxanes and polysiloxanes on the tensile properties of different polymers [43,44]. The highest value was noticed for PLA/1.5%OSS-4OFP:2HEX:2TMOS (25% higher compared to PLA).

Samples containing the OSS-2OFP:6TMOS compound have a tensile strength at a similar level regardless of modifier concentration. The presence of linear alkyl hexyl groups has changed the mechanical behavior of the presented composites causing tensile strength to become more dependent on the concentration of the additive. By comparing the two samples containing an OSS modifier having OFP, TMOS, and HEX groups in its molecule in different proportions, it can be concluded that an increase in the content of OFP groups at the expense of TMOS contributes to an improvement in the tensile properties of PLA. A similar trend of increasing strength for these samples with increasing modifier concentration was maintained in both polymer systems.

**Figure 13:**
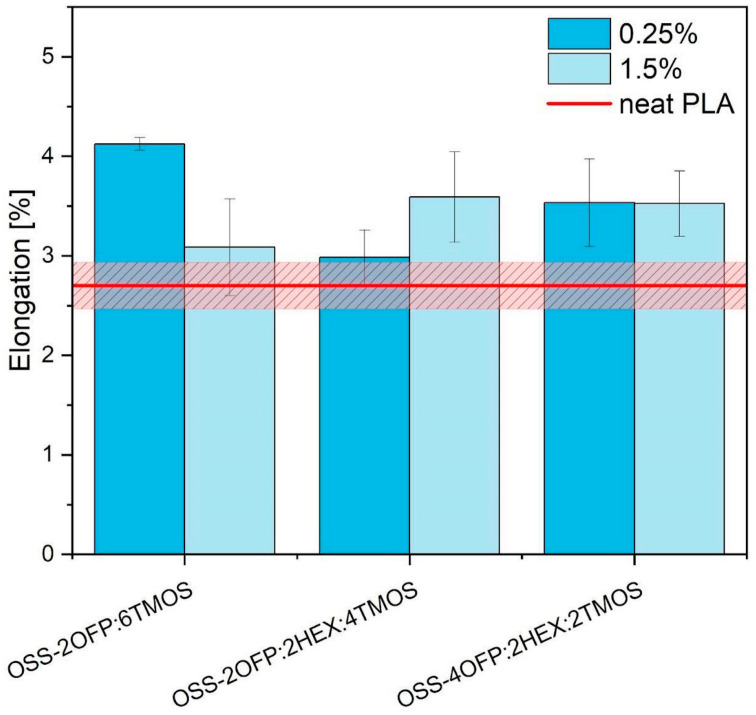
Elongation at maximum load PLA samples in 3D printing.

As confirmed by previous scientific studies, the silsesquioxane additives used to modify PLA present a wide ability to plastify thermoplastic polymers [45]. The elongation at tensile strength experimentally determined for neat PLA is 2.70% ± 0.2% (Figure 13). The increased elastic properties are observed in all PLA composite variants. This is a common effect observed during the plasticization of polymers and was also noted by Chen Yi et al. and Liu Z. et al. in their studies on modifying PLA using organosilicon compounds [46,47]. The 0.25% OSS-2OFP:6TMOS in the PLA improved elongation by nearly 60% compared to PLA. Considering the range of scattered results, the rest of the compounds used show similar effects on the tensile deformation of the printed samples. Plasticizers added to the polymer matrix enhance the spaces between the macromolecules and the mobility of their chains. The small molecules of the modifier (compared to PLA) can move more freely in the polymer matrix due to their size. These small modifier particles can penetrate the polylactide chains easily, effectively increasing the free spaces by separating PLA macromolecules. As the additive concentration of OSS-2OFP:6TMOS increases, a slight decrease in the strain at break was observed (unlike with the other modifiers), possibly due to the interaction of trimethoxysilyl groups, which can cross-link each other [48].

#### 3.7.2. Impact Test

**Figure 14:**
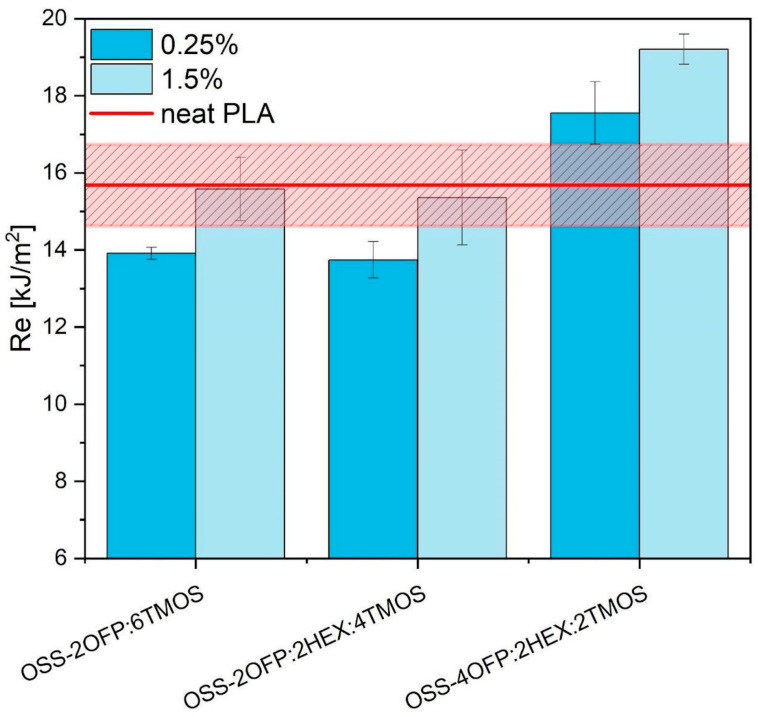
Impact strength of PLA samples in 3D printing.

Charpy impact tests were conducted to assess the influence of the different types of octaspherosilicates on the PLA’s impact properties (Figure 14). The impact strength of neat PLA has a range of 15.7 ± 1.1 [kJ/m^2^]. Among the modifiers described in this paper, the addition of OSS-4OFP:2HEX:2TMOS slightly improves the impact resistance of 3D-printed PLA samples (19.21 ± 0.39 [kJ/m^2^] for 1.5 wt%), which can be correlated with the microstructure of the printed shapes presented in the optical microscopy images in the previous section (Figure 6). The composite samples of PLA/OSS-4OFP:2HEX:2TMOS, with both modifier concentrations, demonstrate a remarkable coherence in interlaminar remelting and a minimal presence of free volumes in contour layers (Figure 6D1). High-quality interlaminar remelting eliminates notches in the sample, which are responsible for the weakening of the structure being the point of origin of the cracking causing the samples to exhibit higher impact strength (an increase of 20% compared to PLA). The largest spaces, both between the outer contours and between the contour and infill of the sample, are found in the OSS-2OFP:2HEX:4TMOS composite (Figure 6C2) leading to the lower impact strength of these samples. The investigation of the microstructure reveals a clear correlation between the concentration of the modifier in the composite and the extent of interlayer remelting, which is directly reflected in the increase in impact strength.

### 3.8. Rheology

**Figure 15:**
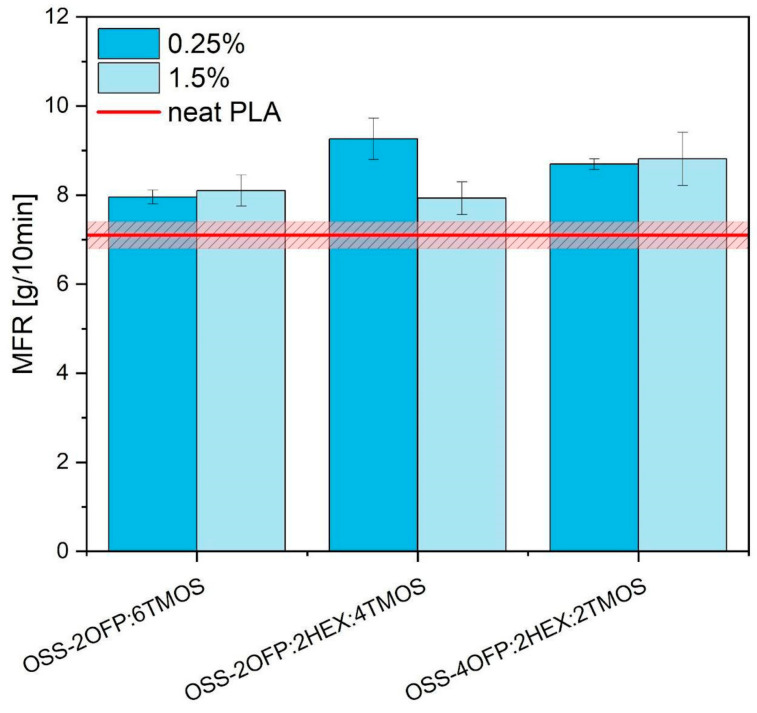
Melt flow rate of PLA composites.

The melt flow rate (MFI) of neat PLA is 7.1 ± 0.3 [g/10min]. The addition of modifiers containing hexyl groups at concentrations of 0.25% and 1.5% significantly increases the MFI for PLA composites (Figure 15). The highest MFI values were achieved for the composite modified with OSS-2OFP-2HEX-4TMOS, showing an increase of 31% compared to neat PLA. This substantial improvement in the MFI indicates that the addition of these specific modifiers enhances the processability of PLA composites.

Moreover, the increase in MFI suggests a pronounced plasticizing effect of the modifiers and facilitates easier flow under processing conditions. This plasticizing effect is crucial for applications requiring lower processing temperatures and improved material flexibility. This positive impact on processing is further corroborated by microscopic images presented in Section 3.2, which illustrate more homogeneous composites. The enhanced flow properties facilitate better molding and extrusion processes, potentially leading to higher-quality end products.

## 4. Conclusions

This study successfully demonstrates the tailoring of hydrophobicity and anti-ice adhesive properties in 3D-printed polylactide (PLA) materials through the incorporation of organosilicon compounds. The addition of fluorinated octaspherosilicate derivatives significantly increased water contact angles, affirming the successful hydrophobic modification of PLA. Hydrophobic surfaces were achieved using modifiers with highly hydrophobic fluoroalkyl/alkyl groups. The highest contact angle measured was 97.8° for the system modified with OSS-2OFP:2HEX:4TMOS. If the fluorinated part (OFP) were to be removed and only the aliphatic portion retained, the overall effect would likely not be fully comparable. While modifiers with long-chain alkyl groups do increase surface hydrophobicity, the unique properties of fluorinated groups result in a significantly stronger effect. Fluorinated chains provide not only enhanced hydrophobicity but also oleophobicity, which contributes to a broader range of repellency, including resistance to oils and other non-polar substances.

Mechanical testing revealed that the incorporation of organosilicon additives did not compromise the tensile strength of PLA and, in some instances, improved impact resistance, particularly with the OSS-4OFP:2HEX:2TMOS additive.

Differential scanning calorimetry (DSC) analysis confirmed the effectiveness of organosilicon modifiers as nucleating agents, which was observed through a lower cold crystallization temperature (up to 16.1 °C).

The study suggests that organosilicon-modified PLA can broaden the material’s application scope by producing moisture and ice-resistant 3D-printed prototypes for various industrial applications, thereby enhancing the durability and versatility of 3D-printed components. Overall, these findings indicate that the modification of PLA with organosilicon compounds is a promising approach to enhancing the material’s performance for advanced applications, potentially leading to its broader adoption in various industries.

## 5. Patents

B. Sztorch, R.E. Przekop, R. Konieczna, D. Pakuła, J. Głowacka, B. Marciniec, Polish Patent pending no: P.445796.

## Figures and Tables

**Table 1:** Amounts of olefins used in the reactions.

Code	Amount of OFP/g	Amount of TMOS/g	Amount of HEX/g
2OFP:6TMOS	13.61	17.46	-
2OFP:2HEX:4TMOS	13.61	11.64	3.30
4OFP:2HEX:2TMOS	27.22	5.82	3.30

**Table 2:** The parameters of the 3D printing.

Properties	Parameters
Nozzle diameter	0.4 mm
Extruder temperature	190 °C
Bed temperature	60 °C
Layer height	0.18 mm
Bottom and top layers number	3
Fill style	linear
Fill angle	45°
Infill density	100%
Printing speed	80 mm/s

**Table 3:** Results of thermal analysis of neat PLA and composites.

Code	Temperature at 1% Weight Loss/℃	Temperature at 5% Weight Loss/℃	Onset Temperature/°C	Temperature at Maximum Weight Change Rate/℃
Neat PLA	253.2	328.5	342.9	362.4
PLA + 1.5% OSS-2OFP:6TMOS	293.5	326.9	342.3	359.4
PLA + 1.5% OSS-2OFP:2HEX:4TMOS	296.8	328.3	342.6	360.0
PLA + 1.5% OSS-4OFP:2HEX:2TMOS	293.6	321.1	336.2	358.7

**Table 4:** Determined temperatures and crystallinity based on DSC.

	First Cycle	Second Cycle	First Cycle	Second Cycle	First Cycle	Second Cycle	First Cycle	Second Cycle
	Tg	Tg	Tcc	Tcc	Tm	Tm	Degree of Crystallinity/%	Degree of Crystallinity/%
Neat PLA	64.7	62.4	124.3	123.1	155.2	153.2	14.5	5.4
PLA + 1.5% OSS-2OFP:6TMOS	65.7	62.2	123.9	109.0	153.8	153.4	13.6	28.9
PLA + 1.5% OSS-2OFP:2HEX:4TMOS	62.3	62.2	121.0	107.0	151.3	152.2	23.7	24.9
PLA + 1.5% OSS-4OFP:2HEX:2TMOS	65.4	62.1	108.2	108.8	154.2	149.5	27.6	27.4

**Table 5:** Water contact angle.

	Surface Character	Concentration/%	Contact Angle/°
Neat PLA	*Hydrophilic*	*-*	70.2 ± 1.2
OSS-2OFP:6TMOS	*Hydrophobic*	0.25	84.6 ± 0.7
		1.5	94.8 ± 1.1
OSS-2OFP:2HEX:4TMOS	*Hydrophobic*	0.25	91.1 ± 1.9
		1.5	97.8 ± 1.0
OSS-4OFP:2HEX:2TMOS	*Hydrophobic*	0.25	94.3 ± 1.1
		1.5	90.7 ± 0.2

## Data Availability

The original contributions presented in the study are included in the article, further inquiries can be directed to the corresponding author.
